# Supplementary material for: Human ARF Specifically Inhibits Epimorphic Regeneration in the Zebrafish Heart
Source: Genes (Basel). 2020 Jun 18;11(6):666. doi: 10.3390/genes11060666 (PMC7349231; doi:10.3390/genes11060666)
Supplement: Supplementary file 1 [file genes-11-00666-s001.pdf]

SUPPLEMENTARY MATERIALS

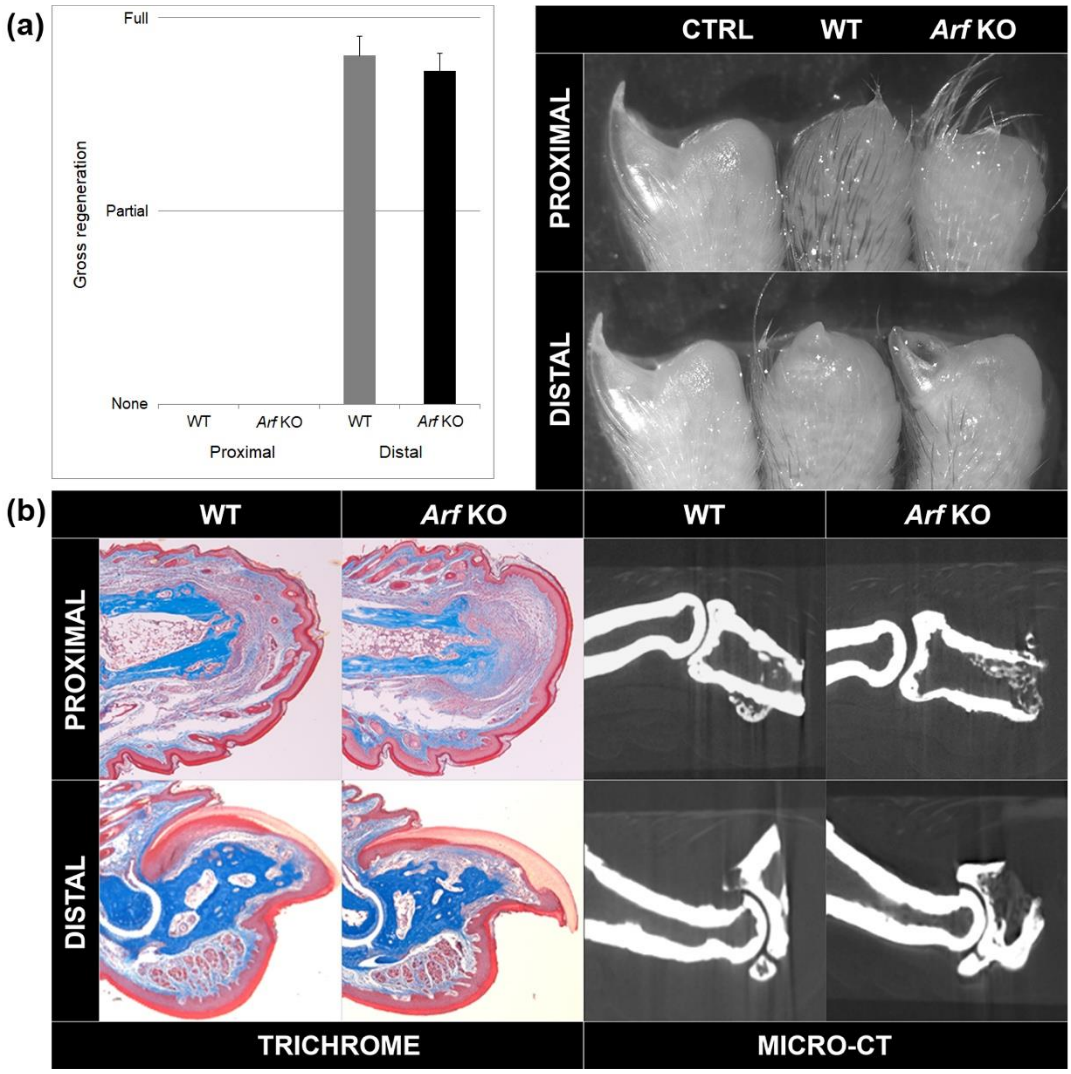

Figure S1. *Arf* deficiency is insufficient for murine digit regeneration. (a) Gross morphological regeneration of digits at 50 days post-amputation (dpa). The graph shows that for samples collected between 20-60 dpa, both WT and *Arf* deficient mice had no regeneration in digits amputated proximal to the nail bed and nearly full regeneration in digits amputated distal to the nail bed. N = 12 mice. Gross anatomy of digits is visualized at 20 dpa. (b) Histology of digits at 60 dpa and micro-CT of digits at 20 dpa. Histology of proximal amputation shows soft tissue epithelialization but no epimorphic regeneration of any nail organ components in both WT and *Arf* deficient mice. Histology of distal amputation shows fully recovered nail in both WT and *Arf* KO mice. Micro-CT of the proximally amputated margin for WT shows no callus, mature ossification, or cortication. Focal osseous proliferation is noted proximal to the amputation site. Comparatively in *Arf* deficient digits, intramedullary sclerosis is noted at the amputation margin, and disorganized periosteal osseous proliferation or callus is noted proximally and extending to the amputation margin. However, no mature ossification or cortication is noted at the amputation margin, and no ossification is noted distal to the amputation margin. Micro-CT of distally amputated margins for both WT and *Arf* deficient digits show no callus, mature ossification, or cortication.
